# A Phase I/IB, Open Label, Dose Finding Study to Evaluate Safety, Pharmacodynamics and Efficacy of Pembrolizumab in Combination With Vorinostat in Patients With Advanced Prostate, Renal or Urothelial Carcinoma

**DOI:** 10.1002/cam4.70725

**Published:** 2025-03-27

**Authors:** Roberto Pili, David I. Quinn, Nabil Adra, Theodore Logan, Sean Colligan, Heather N. Burney, Noah M. Hahn

**Affiliations:** ^1^ University at Buffalo Buffalo New York USA; ^2^ USC Norris Comprehensive Cancer Center Los Angeles California USA; ^3^ Indiana University Melvin and Bren Simon Comprehensive Cancer Center Indianapolis Indiana USA; ^4^ Indiana University Department of Biostatistics and Health Data Science Indianapolis Indiana USA; ^5^ The Sidney Kimmel Comprehensive Cancer Center at Johns Hopkins University Baltimore Maryland USA

**Keywords:** genitourinary malignancies, histone deacetylase inhibitor, PD‐1 inhibitor

## Abstract

**Background:**

Immunosuppressive factors such as regulatory T cells and myeloid‐derived suppressive cells (MDSCs) limit the efficacy of immunotherapies. Histone deacetylase (HDAC) inhibitors have been shown to have immunomodulatory effects. Thus, we conducted a Phase Ib clinical study with the HDAC inhibitor vorinostat and the PD‐1 inhibitor pembrolizumab in patients (pts) with metastatic urothelial (UC), renal (RCC) and prostate (PCA) carcinoma.

**Methods:**

The phase I portion consisted of two dose levels of vorinostat (100 and 200 mg, PO daily 2 weeks ON and 1 week OFF) and a fixed dose of pembrolizumab (200 mg IV every 21 days). Patients (pts) were assigned to three cohorts: Cohort A (previously treated, anti‐PD1/PD‐L1 naïve UC and RCC), Cohort B (previously treated, anti‐PD1/PD‐L1 resistant UC and RCC pts), and Cohort C (PCA pts).

**Results:**

Dose levels 1 and 2 were completed without DLTs. We have enrolled 44 pts. (36 evaluable) in the dose expansion cohorts, and the most common resolved grade 3/4 toxicities were diarrhea, hypophosphatemia, acute kidney injury, anemia, and hypothyroidism. For Cohort A (13 pts), B (11 pts), and C (12 pts) the objective response rate was 8%, 0%, and 17%, and the median progression‐free survival was 2.9, 3.5, and 3.5 months, respectively. Four partial responses were observed, and two PCA pts. had a complete biochemical response with undetectable PSA. Persistent lower levels of peripheral CD11^+^, CD14^+^ HLA‐DR^−^ monocytic MDSCs were associated with clinical benefit.

**Conclusion:**

The combination of vorinostat and pembrolizumab is relatively well tolerated and may be active in a subset of immune checkpoint‐resistant UC/RCC pts. and immune checkpoint‐naïve PCA pts.

**Trial Registration:** NCT02619253

## 1 Introduction

Immune checkpoint inhibitors (ICIs) have dramatically changed the treatment options for several solid tumor types, including urothelial carcinoma (UC) and renal cell carcinoma (RCC) [1, 2, 3]. ICIs have been shown to have anti‐tumor activity in clear cell RCC patients both in first‐ and second‐line therapies [4, 5, 6]. Both PD‐1 and PD‐L1 inhibition have demonstrated anti‐tumor activity and clinical responses in UC patients who were either cisplatin‐ineligible or had progressed while on a cisplatin treatment regimen [7]. In contrast, the activity of ICIs on unselected PCA patients has been anecdotal. However, despite the observed success of these treatments in some patients, several patients do not achieve durable responses to ICIs [8]. Thus, there is a critical need to develop novel combination treatments to overcome mechanisms of acquired/intrinsic resistance. Several reports suggest that the clinical response to ICIs might be driven by the immunosuppressive microenvironment through the recruitment of T regulatory cells (Tregs) and myeloid‐derived suppressor cells (MDSCs) [9, 10, 11]. Thus, the identification of potential signatures predicting a more likely response to ICIs may help to identify early on which patients might benefit the most and which ones should be switched to alternative therapies.

Histone deacetylase (HDAC) inhibitors induce acetylation of several histone and non‐histone proteins, which contribute to a broad spectrum of both anti‐tumor and immunomodulatory activity of these agents. Pan HDAC inhibitors have demonstrated both immunosuppressive and immunopromoting properties by modulating the expression of cytokines by innate and adaptive immune cells, augmenting the activity of macrophages and dendritic cells, and affecting the expression of major histocompatibility (MHC) molecules on antigen presenting cells (APCs) [12, 13]. Several groups, including ours, have demonstrated that HDAC inhibitors can deplete or diminish the immunosuppressive functions of Tregs and MDSCs, which has been shown to augment effector T cell function and improve responses to a wide spectrum of immunotherapies both pre‐clinically and in patients with solid tumors [9, 11, 13]. Specifically, our laboratory has demonstrated that the class I HDAC inhibitor entinostat suppresses Treg cell function, enhances immune responses, and increases the antitumor effect of immunotherapies in preclinical models of kidney and prostate cancer [14, 15, 16, 17].

To test the hypothesis that HDAC inhibition may enhance the efficacy of ICIs in the clinic, we have conducted a phase I/Ib, open label, dose finding study of the combination of the Class I/II HDAC inhibitor vorinostat and the PD‐1 inhibitor pembrolizumab in patients with advanced RCC, urothelial and prostate cancer. We collected patient PBMCs both prior to treatment, and post treatment once per month for the duration of the patients' participation on the trial and performed analyses by flow cytometry to identify potential predictive markers of response.

## 2 Materials and Methods

### 2.1 Study Design and Objectives

**FIGURE 1:**
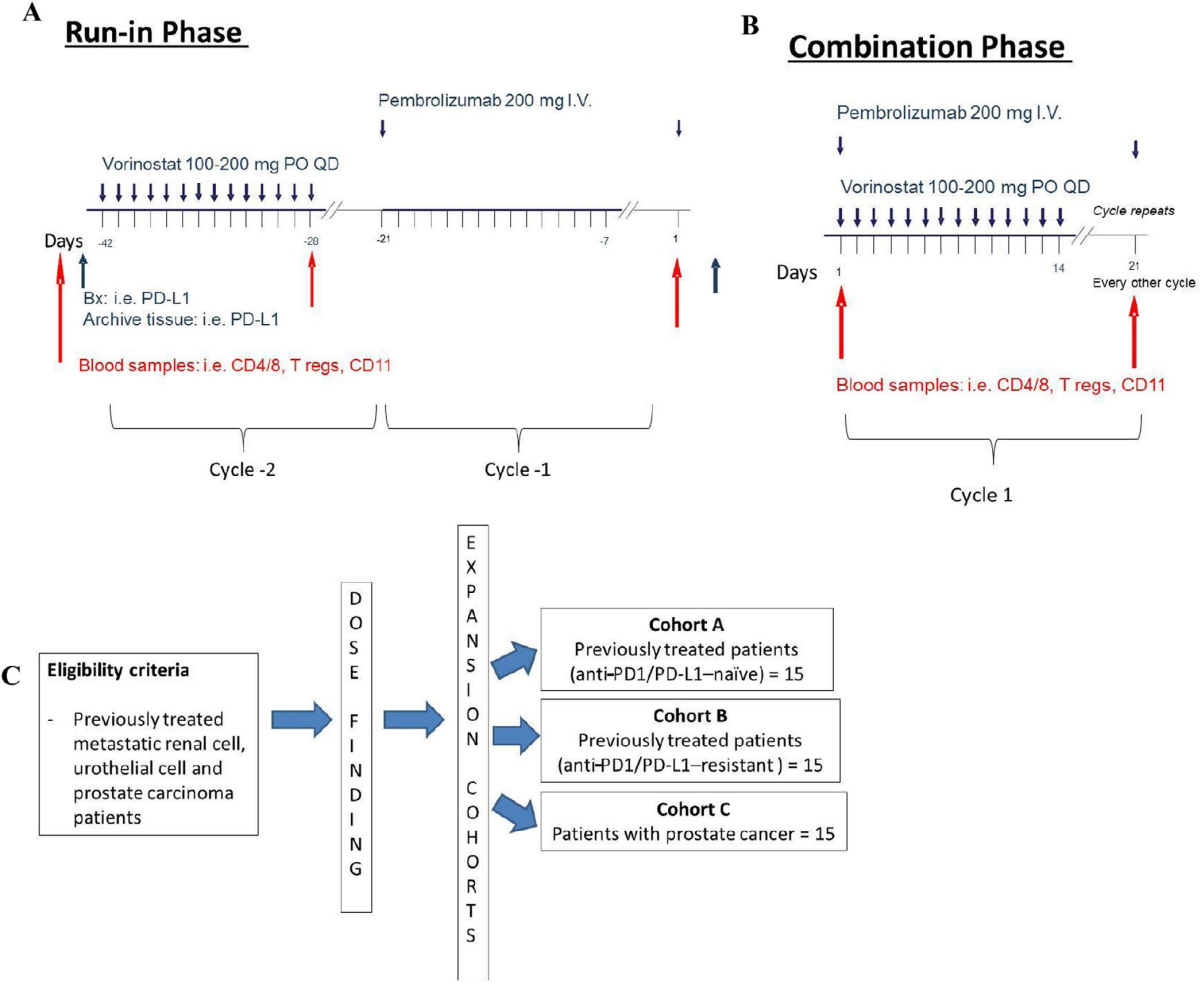
Study schema. (A) During the Run‐in Phase, patients who decided to participate were exposed to single drugs (cycle −2 and cycle −1) before being treated with the combination (cycle 1) (B). (C) Expansion cohorts included UC and RCC patients with no prior IOs (Cohort A), with prior ICIs (Cohort B), and PCA patients (Cohort C).

This was an investigator‐initiated, multi‐site, Phase I/IB clinical trial conducted at the Indiana University Melvin and Bren Simon Comprehensive Cancer Center (Indianapolis, IN), the University of Southern California Norris Comprehensive Cancer Center (Los Angeles, CA), and the Sidney Kimmel Comprehensive Cancer Center at Johns Hopkins (Baltimore, MD). The study was approved by the institutional review boards (IRBs)/Ethics Committees at Indiana University Melvin and Bren Simon Comprehensive Cancer Center, the University of Southern California Norris Comprehensive Cancer Center, and the Sidney Kimmel Comprehensive Cancer Center at Johns Hopkins. The clinical trial registration identifier was NCT02619253. The study included a run‐in phase where patients were exposed to sequential monotherapy cycles with vorinostat first and then pembrolizumab before receiving the combination to assess the potential impact of the single agents on the immune cells (Figure 1A,B). Participation in the monotherapy run‐in phase was optional, and only a fraction of patients decided to participate. The dose‐finding portion was conducted according to an escalation dose design of vorinostat. In Dose Level 1, patients received pembrolizumab at 200 mg IV Q3W and vorinostat 100 mg PO QD × 14 days. In Dose Level 2, patients received pembrolizumab at 200 mg IV Q3W and vorinostat 200 mg PO QD × 14 days. The primary endpoint of the study was to determine the safety and tolerability of vorinostat in combination with pembrolizumab in patients with metastatic renal, urothelial, and prostate cancer. In the expansion cohort, the recommended Phase II dose (Dose Level 2) was tested. Patients received pembrolizumab at 200 mg IV Q3W and vorinostat 200 mg PO QD × 14 days. The expansion cohort was split into three distinct groups: Cohort A: previously treated renal and urothelial cancer patients (anti‐PD1‐naïve); Cohort B: previously treated renal and urothelial patients (anti‐PD1‐resistant); Cohort C: prostate cancer patients (Figure 1C). Anti‐PD1‐resistant disease was defined as progression after any prior anti‐PD1 therapy.

### 2.2 Patient Eligibility

Following written informed consent, histologically confirmed metastatic or unresectable renal cell, urothelial, or prostate carcinoma was a requirement for enrollment. Patients (except for prostate cancer) were required to have measurable disease by RECIST 1.1 criteria [18]. Tumor response in prostate cancer patients was assessed by PCWG criteria. ECOG performance status ≤ 1 and a life expectancy of greater than 6 months were required. Normal organ and marrow function were required. Key exclusion criteria included concurrent treatment with other investigational agents or prior treatment with HDAC inhibitors, including valproic acid; active or untreated CNS metastases or malignancies other than non‐melanoma skin cancers. For more exclusion criteria, please refer to our previous study with vorinostat [19].

### 2.3 Pre‐Treatment Assessment

Within 1 week prior to the start of treatment, patients had routine visits including physical exams and laboratory tests. The imaging modalities for tumor assessment included CT, bone scan, and MRI, which were performed within 30 days as baseline and were repeated every 6 weeks.

### 2.4 Treatment

Each cycle of therapy was given every 21 days. Oral vorinostat (100 or 200 mg) was taken daily for 14 days with 7 days holding, and pembrolizumab (200 mg) was administered intravenously on Day 1 of each cycle. The schedule of vorinostat with 7 days off therapy was based on our previous experience with this HDAC inhibitor [19]. Patients were assessed for tumor response every 2 cycles or 6 weeks. The treatment continued until disease progression, intercurrent illness that prevented further administration of treatment, unacceptable adverse event(s), or the patient decided to withdraw from the study. Patients were seen 28 days following the last dose administration for toxicity assessment and then every 3 months or until death.

### 2.5 Dose Modifications

The starting dose of vorinostat was 100 mg orally QD (Dose Level 1) per day for days 1 through 14. If dose limiting toxicity (DLT), defined as any grade 3 or 4 hematological/non‐hematological toxicities attributable to vorinostat and/or pembrolizumab or any toxicity responsible for treatment delay for > 2 weeks, occurred during the first cycle (first 21 days) at dose level 2, the patient dose level was de‐escalated to dose level 1 (100 mg QD). The dose of pembrolizumab was 200 mg IV on day 1, every 21 days.

### 2.6 Toxicity and Response Evaluation

Toxicities were assessed as per CTCAE v4 and reported utilizing descriptive statistics. In Phase Ib, the anti‐tumor response was assessed utilizing the RECIST criteria. The primary efficacy endpoints of the phase Ib portion of the study were the objective response rate (ORR), defined as the proportion of patients with PR or better, and PFS, defined as no evidence of either clinical or anatomic progression. For the analysis of the planned correlative studies, patients were classified as Responders if they achieved an objective response or stable disease for at least 6 months, and Progressors if they had progressive disease as the best response.

### 2.7 Flow Cytometric Analysis of Patient PBMCs

Patient PBMCs were isolated, and RBCs were removed by ACK lysis. Cells were FC blocked using anti‐CD16/32 antibody in flow cytometry buffer (DPBS+2% FBS + 0.5 mM EDTA) before being stained with surface markers. Cells were then stained for intracellular proteins (eBioscience FOXP3/Transcription Factor Staining Buffer set; Cat# 00–5523‐00). Specific antibodies were utilized to characterize the different immune subsets (Figure S1). Stained samples were analyzed using a BD FACS Fortessa flow cytometer running BD FACSDiva software. Results were analyzed using De Novo FCS Express Flow Cytometry Software, and statistics were quantified using Graphpad.

### 2.8 Statistical Considerations

Categorical data are reported, including frequency and percentage. Continuous measures were summarized by mean and standard deviation. ORR was estimated as the proportion of patients with PR or better, and a binomial exact 95% confidence interval was reported for each expansion cohort. The Kaplan–Meier method was used to estimate median PFS as determined from the start date of treatment to the date of progression for patients who progressed or the date of death. The observations of patients remaining alive and progression‐free were censored at the date of the last disease evaluation. The median and a 95% confidence interval were provided along with a Kaplan–Meier plot with the number of patients at risk. Patients considered evaluable for the primary efficacy objectives were in the expansion cohorts and had at least one follow‐up assessment of disease status after completing 1 cycle of combination therapy. Treatment‐related toxicities were reported for all patients who received at least one dose of study drugs. Toxicities were classified as possibly, probably, or definitely related to treatment, and were reported by cohort and drug. All analyses were performed using SAS Version 9.4 (Cary, NC).

## 3 Results

### 3.1 General

The study began accrual in March 2017, and the last patient enrolled was in September 2019. The study closed in March 2023. Fifty‐two patients were enrolled, and 52 were evaluable for safety and 36 for efficacy, as shown in the CONSORT diagram (Figure S2). The clinical characteristics are summarized in Table 1. Based on the ECOG performance status, most patients were classifiable as intermediate risk [20].

**TABLE 1:** Baseline demographics summary.

Characteristic	Dose finding	Cohort A	Cohort B	Cohort C
Number of participants, *n*	8	15	14	15
Age, mean ± standard deviation	67.7 ± 9.7	64.3 ± 8.6	65.4 ± 10.3	69.0 ± 6.5
Age category, *n* (%)				
Between 18 and 65 years	2 (25.0)	6 (40.0)	6 (42.9)	1 (6.7)
≥ 65 years	6 (75.0)	9 (60.0)	8 (57.1)	14 (93.3)
Gender, *n* (%)				
Female	4 (50.0)	3 (20.0)	4 (28.6)	0 (0.0)
Male	4 (50.0)	12 (80.0)	10 (71.4)	15 (100.0)
Race, *n* (%)				
White	8 (100.0)	13 (86.7)	14 (100.0)	13 (86.7)
Black or African American	0 (0.0)	0 (0.0)	0 (0.0)	2 (13.3)
Other	0 (0.0)	2 (13.3)	0 (0.0)	0 (0.0)
Ethnicity, *n* (%)				
Non‐Hispanic	8 (100.0)	13 (86.7)	13 (92.9)	15 (100.0)
Hispanic	0 (0.0)	2 (13.3)	1 (7.1)	0 (0.0)
ECOG performance status, *n* (%)^a^				
0	2 (25.0)	6 (40.0)	4 (30.8)	4 (26.7)
1	6 (75.0)	8 (53.3)	9 (69.2)	10 (66.7)
2	0 (0.0)	1 (6.7)	0 (0.0)	1 (6.7)
Disease type, *n* (%)^b^				
Prostate		0 (0.0)	0 (0.0)	15 (100.0)
Renal		7 (58.3)	5 (38.5)	0 (0.0)
Urothelial		5 (41.7)	8 (61.5)	0 (0.0)
Prior lines of therapy, median (min–max)	6 (3–9)	3 (1–16)	7 (1–11)	5 (3–9)

^a^ One patient had a missing ECOG score at baseline.

^b^ No disease information was available for patients in the dose‐finding cohort, and disease type is not known for three patients in Cohorts A and one patient in Cohort B.

### 3.2 Safety and Tolerability

During the dose finding cohorts, in Dose Level 1, four patients (three DLT evaluable) received pembrolizumab at 200 mg IV Q3W and vorinostat 100 mg PO QD × 14 days. For Dose Level 2, four patients (three evaluable) received pembrolizumab at 200 mg IV Q3W and vorinostat 200 mg PO QD × 14 days. During the Expansion Cohort at Recommended Phase II Dose (Dose Level 2) patients received pembrolizumab at 200 mg IV Q3W and vorinostat 200 mg PO QD × 14 days. The expansion cohort was split into three distinct cohorts: (1) Cohort A: previously treated UC and RCC pts. (antiPD1‐naïve) = 15 pts.; (2) Cohort B: previously treated UC and RCC pts. (antiPD1‐resistant) = 14 pts.; (3) Cohort C: PCA pts. = 15pts. Dose levels 1 and 2 were completed without DLTs, and 200 mg was the Phase II recommended dose for vorinostat. The most common treatment‐related toxicities during the dose finding cohorts (8 pts) were nausea/vomiting, anorexia, diarrhea, fatigue, and hyperthyroidism (Table 2). There were no grade 3/4 vorinostat‐related toxicities in either dose finding cohort. The most common grade 3/4 toxicities were acute anemia (*n* = 3), diarrhea (*n* = 2), fatigue (*n* = 2), hyponatremia (*n* = 2) hypothyroidism (*n* = 1) during the dose expansion, where we enrolled 44 pts. evaluable for safety (Table S1). Thirteen patients (29.5%) in the dose expansion cohorts experienced at least one grade 3 or 4 toxicity.

**TABLE 2:** Overall treatment‐related toxicities—dose finding cohort.

CTCAE term	Grade 1	Grade 2	Grade 3	Grade 4	Total	Total percent	Grade 3–4	Grade 3–4 percent
Nausea	2	1	0	0	3	37.50	0	0.00
Anorexia	0	2	0	0	2	25.00	0	0.00
Diarrhea	2	0	0	0	2	25.00	0	0.00
Fatigue	0	2	0	0	2	25.00	0	0.00
Hyperthyroidism	1	1	0	0	2	25.00	0	0.00
Vomiting	2	0	0	0	2	25.00	0	0.00
Alopecia	1	0	0	0	1	12.50	0	0.00
Anemia	0	1	0	0	1	12.50	0	0.00
Aspartate aminotransferase increased	1	0	0	0	1	12.50	0	0.00
Cardiac troponin I increased	1	0	0	0	1	12.50	0	0.00
Cough	0	1	0	0	1	12.50	0	0.00
Dizziness	1	0	0	0	1	12.50	0	0.00
Dry mouth	1	0	0	0	1	12.50	0	0.00
Gastroesophageal reflux disease	1	0	0	0	1	12.50	0	0.00
Hypothyroidism	1	0	0	0	1	12.50	0	0.00
Muscle weakness trunk	0	1	0	0	1	12.50	0	0.00
Pain	0	1	0	0	1	12.50	0	0.00
Sinus tachycardia	0	1	0	0	1	12.50	0	0.00

*Note:* There were no grade 3/4 vorinostat‐related toxicities in either dose finding cohort.

### 3.3 Clinical Activity

**FIGURE 2:**
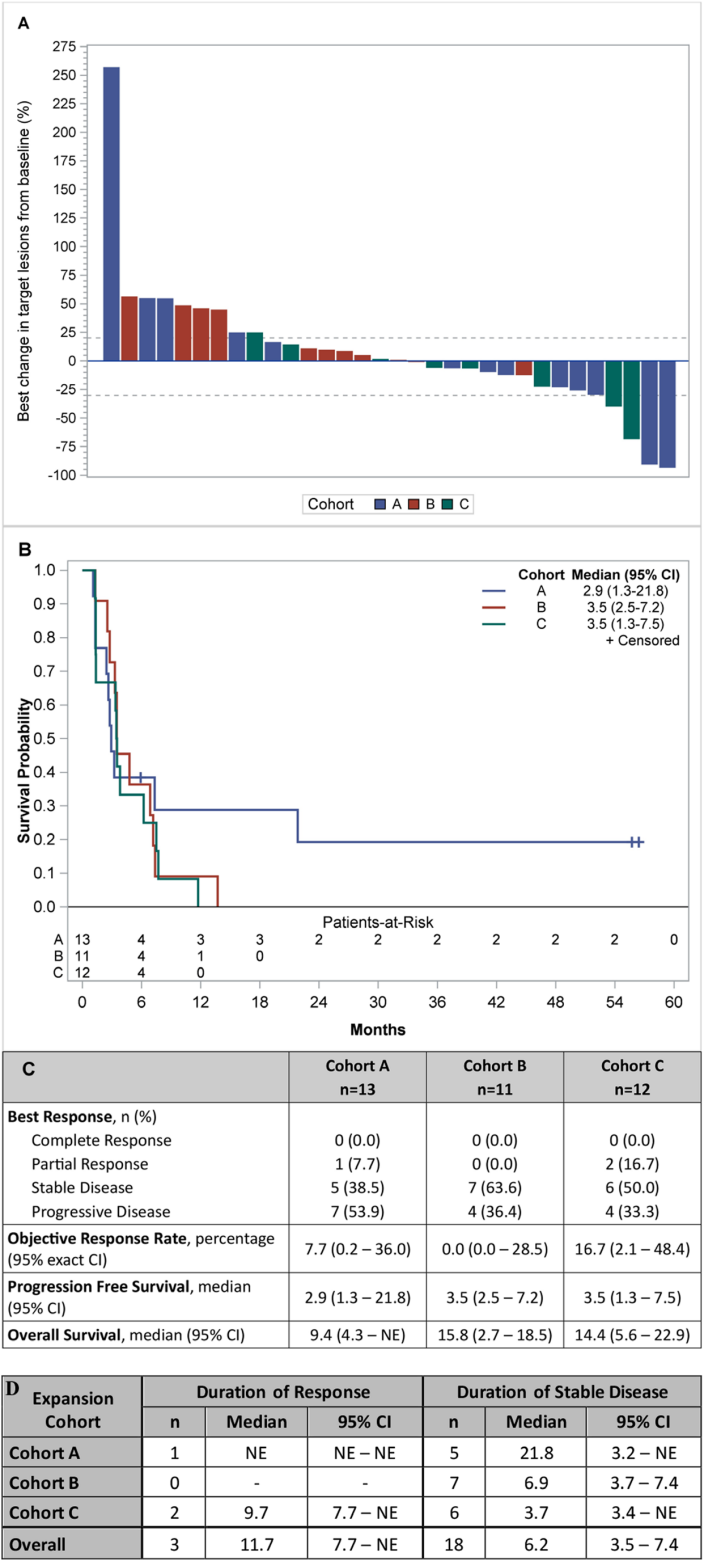
Clinical benefit of vorinostat and pembrolizumab in renal, urothelial, and prostate cancer patients. (A) Waterfall plot with best responses across the different cohorts; (B) progression‐free survival analysis; (C) best response and survival summary statistics; (D) duration of response and duration of stable disease (months).

**FIGURE 3:**
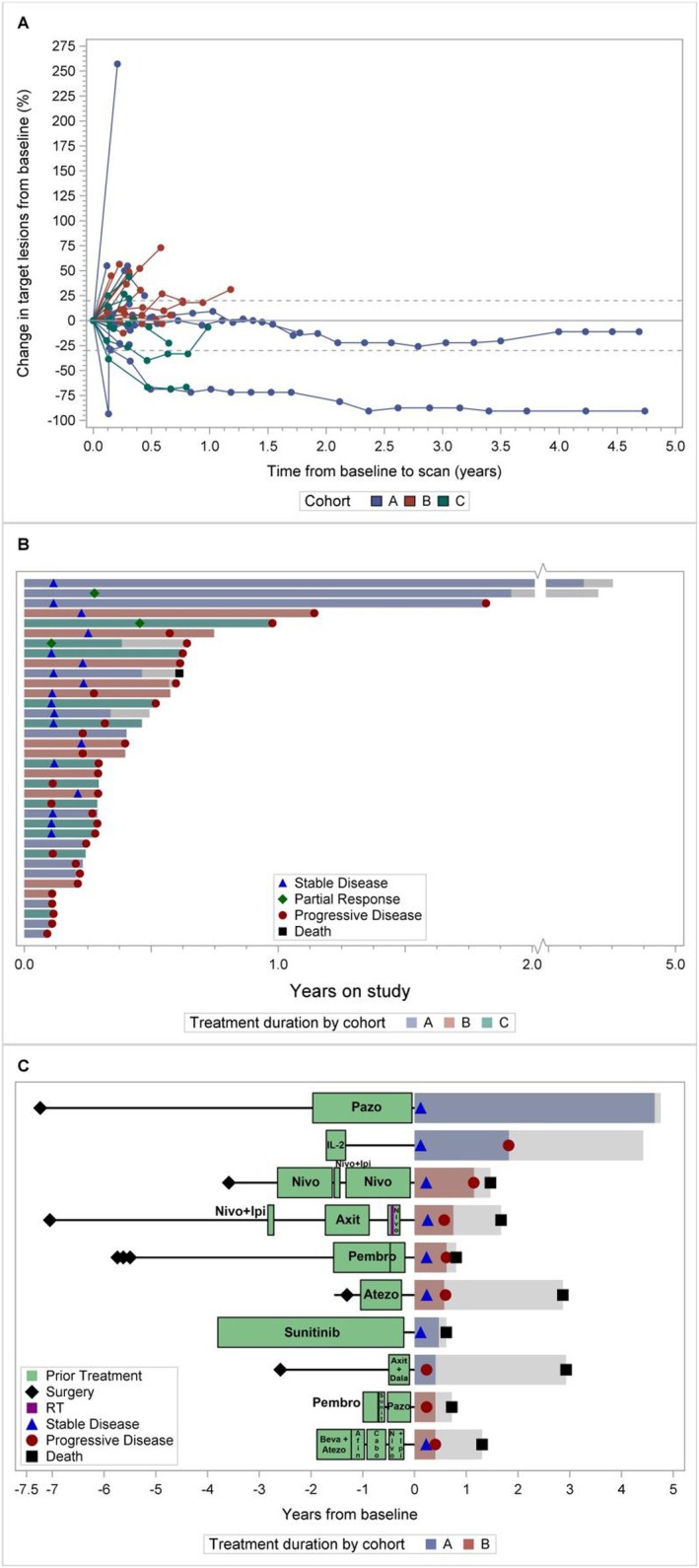
Duration of responses and prior treatments. (A) Spider plot and (B) Swimmer plot. (C) Treatment duration according to prior regimens. The ten patients who were on treatment the longest and had at least one regimen are included in this plot. afin = afinitor, atezo = atezolizumab, axit = axitinib, axit/dala = axitinib/dalantercept, beva/atezo = bevacizumab/atezolizumab, cabo = cabozantinib, ipi/nivo = ipilimumab/nivolumab, nivo = nivolumab, pazo = pazopanib, pembro = pembrolizumab, sunit = sunitinib.

The therapeutic effect of vorinostat and pembrolizumab combination is reported in Figure 2A–D. During the dose finding (6 pts) we observed one objective partial response (PR). Two additional PRs were observed: one patient was eventually not evaluable because deemed ineligible but continued treatment; another pt. had confirmed PR based on irRC but not RECIST. During the expansion cohorts, we observed one objective partial response (1/13 or 7.7%) in Cohort A and two objective partial responses (2 out of 12 or 16.7%) in Cohort C. The two PCA responders also had complete PSA response. The median PFS for Cohort A, B, and C was 2.9 months (95% C.I 1.3–21.8), 3.5 months (95% C.I. 2.2–7.2), and 3.5 months (95% C.I. 1.3–7.5), respectively. Eighteen pts. (50%) had stable disease, including two pts. with durable response for more than 4 years (Figure 3A,B). We did observe stable disease irrespective of the number of prior therapies pts. had received (Figure 3C).

### 3.4 Correlative Studies

**FIGURE 4:**
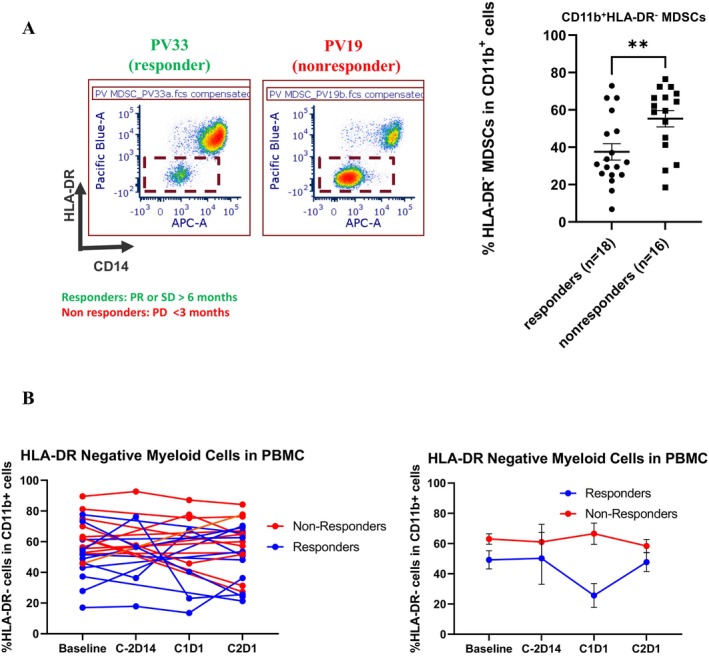
Correlative studies. (A, B) PBMCs were isolated from pts. at both baseline (B) and treatment cycle 2 day 1 (C2). Patients were stratified into 2 groups based on responder status, with responders having received > 6 months of treatment without tumor progression, and non‐responders having exhibited tumor progression within 3 months of treatment start. Pt PBMCs were analyzed for both surface and intracellular markers by flow cytometry. (A) Left: Representative dot plots demonstrating levels of HLA‐DR^−^CD11b^+^CD14^−^ PMN‐MDSCs in the peripheral blood of 1 responder and 1 non‐responder. Right: Quantification of HLA‐DR^−^CD11b^+^ total MDSCs in the peripheral blood of responders and non‐responders at baseline. (B) Changes of HLA‐DR^−^CD11b^+^ total MDSCs in the peripheral blood of responders and non‐responders from baseline through cycle 2 day 1. Data are represented as the mean ± SEM of *n* = 18 responders and *n* = 16 non‐responders. **p < 0.01, by unpaired *t* test.

To assess whether the response to vorinostat and pembrolizumab was associated with a specific immunological signature, we conducted a limited analysis of PBMC at baseline and after 2 cycles of therapy. No statistically significant differences were seen in Tregs and Granzyme B T cells. However, we observed that responders presented with lower levels of CD11b^+^CD14^+^HLA‐DR^−/−^ monocytic MDSCs at baseline as compared to non‐responders (Figure 4A). Interestingly, lower levels were maintained during the treatment (Figure 4B).

## 4 Discussion

Despite the significant advances in the treatment of UC and RCC with several ICIs available, recurrent or progressive disease remains a major hurdle in these diseases. Based on the potential immunomodulatory activity of HDAC inhibition, this clinical trial tested the combination of a pan HDAC inhibitor with the PD‐1 inhibitor pembrolizumab in treatment‐naïve and treatment‐resistant patients with UC or RCC, and then it expanded also to PCA pts. Overall, the toxicities induced by the combination of vorinostat and pembrolizumab did not raise concerns. No DLTs were observed in the dose‐finding cohort. The most common non‐hematologic (anorexia, dehydration, diarrhea, and fatigue) and hematologic (anemia and thrombocytopenia) toxicities were rapidly reversible following drug interruption. The use of potential immunomodulators in combination with immune checkpoint inhibitors always raises the concern for an increased rate of immune‐related side effects. However, the tolerability profile that we have observed confirms the feasibility of combining epigenetic modulators such as HDAC inhibitors with current immunotherapies. The efficacy of this combination was modest, but we observed stable disease in 50% of the pts., including two pts. with a durable response for more than 4 years.

ICIs represent the main stem of treatment for both UC and RCC, ranging from the perioperative setting to the advanced disease. Pembrolizumab is an FDA‐approved drug as a single agent in the adjuvant setting and in combination with receptor tyrosine kinase inhibitors in advanced disease for RCC [21, 22, 23]. Pembrolizumab is also an active drug in UC, and the combination of pembrolizumab with the nectin 4‐targeting antibody drug conjugate enfortumab is the new standard of care in first‐line treatment for metastatic UC patients [24, 25]. Overall, we were disappointed to observe that our combination strategies did not provide greater activity in RCC and urothelial cancer as compared to historical data with a single agent pembrolizumab. The data in Cohort A were particularly disappointing, while there were cases of clinical benefit in Cohort B among pts. with prior ICIs. In our preclinical studies, we have reported that higher doses of pan HDAC (class I and II) inhibitor might have an immune suppressive effect [13]. Thus, there is the possibility that lower doses of vorinostat (50 or 100 mg QD) could have achieved a greater clinical effect. This observation confirms the potential double‐edged sword mechanism underlying the use of epigenetic modulators and the need for greater understanding of the pharmacodynamic implications for this class of agents to fully exploit its potential.

The clinical response that we observed in two PCA pts. was interesting. Vorinostat has been previously reported to have modest activity in patients with PCA [26]. The use of pembrolizumab has also shown some activity in unselected PCA pts., while it has been reported to induce a dramatic response in prostate tumors with high microsatellite instability [27, 28, 29, 30]. We acknowledge that the response we observed could have been just due to pembrolizumab as a single agent. However, a greater understanding of the immune suppressive bone microenvironment and the potential immune modulatory activity of HDAC inhibition might help to develop further this combination strategy for prostate cancer.

Our correlative studies suggest that lower PBMC baseline levels of mononuclear MDSCs (CD11b^+^CD14^+^HLA‐DR^−/−^) were associated with clinical benefit in patients receiving vorinostat and pembrolizumab. However, we can only speculate that the immunophenotype observed in the peripheral blood reflects the tumor microenvironment. Recent reports have suggested the involvement of MDSC accumulation in tumor‐associated immunosuppression in various cancer types [31, 32]. Furthermore, increased MDSC levels have been associated with cancer progression and poor outcomes [33, 34]. Interestingly, studies have also suggested that lower levels of CD11b^+^CD14^+^HLA‐DR^−/low^ at baseline may be associated with a greater response to ICIs [35, 36]. Though we did not observe modulation of this subset following treatment in our patient population, lower levels of these immune suppressive subsets were maintained following treatment with vorinostat and pembrolizumab in patients achieving greater clinical benefit, perhaps contributing to the immune response to this combination strategy.

In summary, our results suggest that vorinostat in combination with pembrolizumab does not enhance the immune‐related toxicities of PD‐1 inhibition but has only a modest activity in a limited number of patients with ICIs resistant UC/RCC and ICIs naïve PCA. We recognize several limitations in our study, including the limited number of patients enrolled and the absence of a comparator arm. Future analysis of epigenetic therapies in combination with immunotherapies will help to determine a subset of UC, RCC, and PCA pts. who could likely have a clinical benefit.

## Author Contributions

**Roberto Pili:** conceptualization (lead), data curation (lead), formal analysis (lead), funding acquisition (lead), investigation (lead), methodology (lead), project administration (lead), supervision (lead), validation (lead), visualization (lead), writing – original draft (lead), writing – review and editing (lead). **David I. Quinn:** data curation (equal), formal analysis (equal), investigation (equal), writing – review and editing (equal). **Nabil Adra:** conceptualization (equal), data curation (equal), formal analysis (equal), writing – review and editing (equal). **Theodore Logan:** data curation (equal), formal analysis (equal), writing – review and editing (equal). **Sean Colligan:** data curation (equal), formal analysis (equal), writing – review and editing (equal). **Heather N. Burney:** data curation (equal), formal analysis (equal), writing – review and editing (supporting). **Noah M. Hahn:** conceptualization (equal), data curation (equal), formal analysis (equal), writing – review and editing (equal).

## Conflicts of Interest

Dr. Roberto Pili has received a research grant from Merck. Dr. Nabil Adra participates in the Data Safety Board Committee of a clinical trial sponsored by Merck. No other disclosures are reported by the other authors.

## Supporting information


Data S1.

## Data Availability

The data that support the findings of this study are available on request from the corresponding author. The data are not publicly available due to privacy or ethical restrictions.
