# Supplementary material for: A Phase I/IB, Open Label, Dose Finding Study to Evaluate Safety, Pharmacodynamics and Efficacy of Pembrolizumab in Combination With Vorinostat in Patients With Advanced Prostate, Renal or Urothelial Carcinoma
Source: Cancer Med. 2025 Mar 27;14(7):e70725. doi: 10.1002/cam4.70725 (PMC11947755; doi:10.1002/cam4.70725)
Supplement: Supplementary file 1 — Data S1. [file CAM4-14-e70725-s001.docx]

**Figure S1 : Antibodies utilized for the immunofluorescence studies**

**Marker**

**Stain/ Fluorescent antibody**

**Myeloid Panel**

Live Dead

Fixable Blue Dead Cell Stain Kit

CD45

PerCP

-

Cy5.5

CD33

PE

-

Cy7

CD11b

PE

CD14

BV605

CD66b

FITC

HLA

-

DR

BV421

Arg1

APC

**Treg** **Panel**

Live Dead

Fixable Blue

Dead Cell Stain Kit

CD45

PerCP

-

Cy5.5

CD3

APC

-

Cy7

CD4

APC

CD8

FITC

CD25

PE

PD1

BV605

FOXP3

BV421

**Granzyme B**

Panel

Live Dead

Fixable Blue Dead Cell Stain Kit

CD45

PerCP

-

Cy5.5

CD3

PE

-

Cy7

CD4

APC

CD8

BV421

Granzyme B

FITC

**Table S1. Grade 3-4 Treatment-related Toxicities – Expansion Cohort**

| **CTCAE Term** | **Grade 3** | **Grade 4** | **Total** | **Total Percent** |
| --- | --- | --- | --- | --- |
| Anemia** | 3 | 0 | 3 | 7 |
| Diarrhea* | 2 | 0 | 2 | 5 |
| Fatigue** | 1 | 1 | 2 | 5 |
| Hyponatremia** | 2 | 0 | 2 | 5 |
| Hypophosphatemia* | 2 | 0 | 2 | 5 |
| Acute kidney injury* | 1 | 0 | 1 | 2 |
| Alanine aminotransferase increased* | 1 | 0 | 1 | 2 |
| Aspartate aminotransferase increased* | 1 | 0 | 1 | 2 |
| Dyspnea* | 1 | 0 | 1 | 2 |
| Edema limbs | 1 | 0 | 1 | 2 |
| Hypokalemia | 1 | 0 | 1 | 2 |
| Hypothyroidism* | 0 | 1 | 1 | 2 |
| Platelet count decreased | 1 | 0 | 1 | 2 |

*Resolved

**One case resolved

**
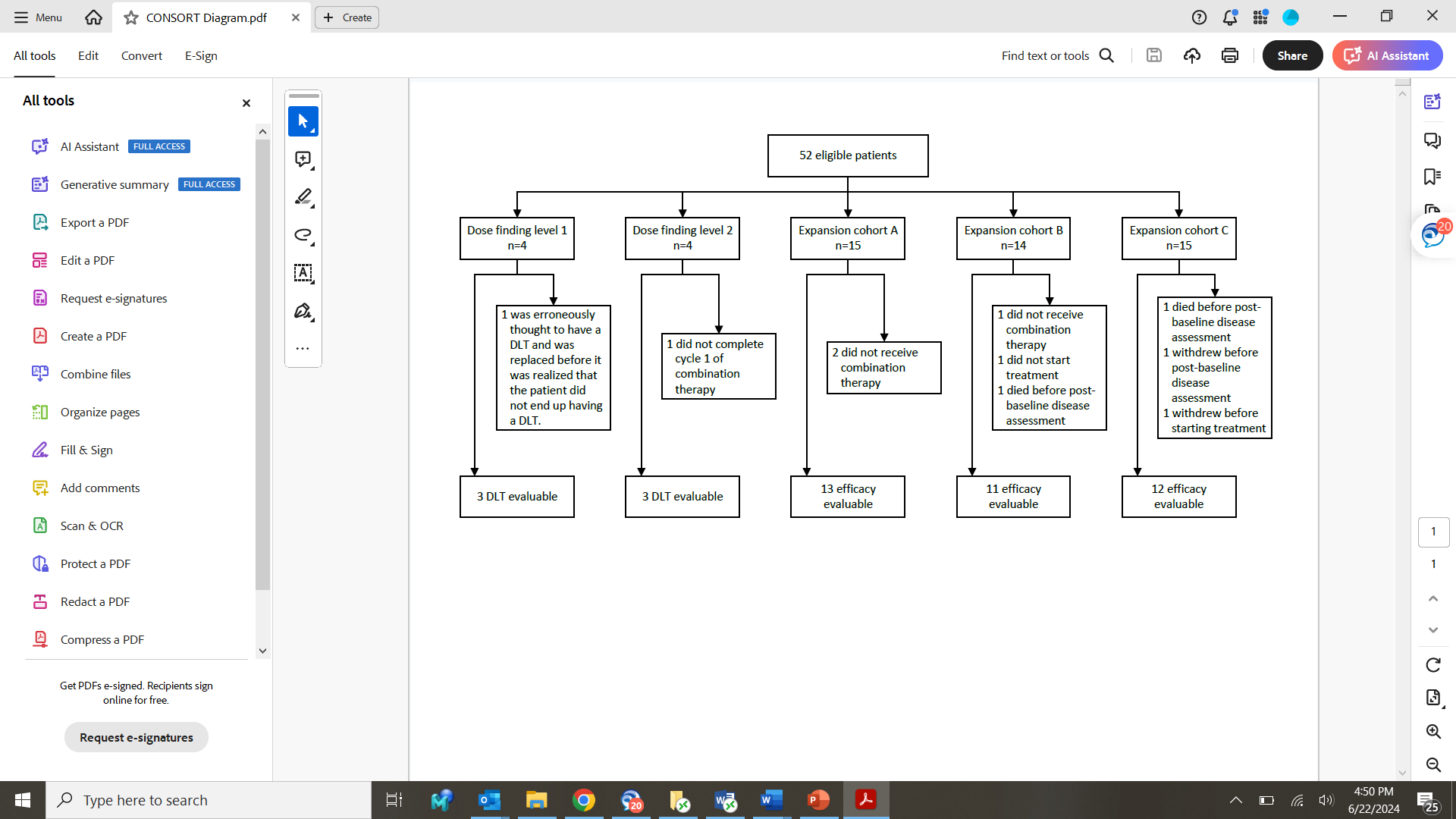
Figure S2 : CONSORT diagram**
